# Contribution to the knowledge of the veterinary science and of the ethnobotany in Calabria region (Southern Italy)

**DOI:** 10.1186/1746-4269-2-52

**Published:** 2006-12-11

**Authors:** Nicodemo G Passalacqua, Giuseppe De Fine, Paolo Maria Guarrera

**Affiliations:** 1 Museo di Storia Naturale della Calabria ed Orto Botanico, Università della Calabria, 87030 Arcavacata di Rende, Italy; 2 via Madonna delle Grazie 9, 88813, Cirò, Italy; 3 Museo Nazionale Arti e Tradizioni Popolari, Piazza Marconi 8-10 00144 Rome, Italy

## Abstract

**Background:**

A series of preliminary research projects on plants used in Calabria (Southern Italy) in veterinary science and in other ethno-botanical fields (minor nourishment, domestic and handicraft sector) was carried out in the last twenty years. From the ethno-botanical point of view, Calabria is one of the most interesting region, since in the ancient times it was subject to the dominant cultures of several people (Greeks, Romans, Byzantines, Arabs, Normans etc.). Until some decades ago the road network was poorly developed and villages were isolated, so that the culture of the "subsistence" and some archaic customs were kept.

**Methods:**

Data were collected by means of "open" interviews to farmers, shepherds and housewives in the last twenty years. More than 100 informants were interviewed, mostly over 50 years old. Plants were identified by local informants through gathering in the area or through examination of the fresh plants collected by the researchers. The collected data were compared with pharmacobotanical papers mainly of southern Italy and with other studies, in order to highlight novelties or concordances of uses.

**Results:**

The use of 62 taxa distributed into 34 families are described. Among these, 8 are or were employed in veterinary science, 8 as anti-parasitic agents, 19 in minor nourishment, 5 as seasoning, 38 for other uses. Some toxic species for cattle are also mentioned.

**Conclusion:**

Among the major findings: the use of *Helleborus bocconei *for bronchitis of bovines and of *Scrophularia canina *for lameness in veterinary science; *Nerium oleander *and *Urginea maritima *as anti-parasitic agents; *Epilobium angustifolium, Centaurea napifolia *L. and *C. sphaerocephala *L. in minor nourishment.

## Background

A research was carried out in some localities of Calabria region (Italy) in the last twenty years on the traditions relevant to the plants used in veterinary science and in other ethno-botanical fields (minor nourishment, domestic and handicraft sector) in order to preserve the historical "memory" of the territory and of the local culture.

The only papers existing on the ethno-botany of Calabria region (mainly on uses in human medicine) are by Leporatti and Pavesi [1] and by Barone [2]; some information is also furnished by Bernardo [3], La Sorsa [4] and Lupia [5]. In the food field, two recent contributions were published by Picchi and Pieroni [6] and by Nebel et al. [7].

Calabria region (15080 km^2^) extends about 250 km north to south in the center of the Mediterranean Sea, bordering with Ionian Sea to east and south, with Tyrrhenian Sea to west, and with Basilicata region to north; the Messina Strait separates Calabria from Sicily. The region is mostly mountainous and about 90% of the surface is occupied by two section of Apennine chain: southern Apennines, calcareous, with Pollino Massif (Serra Dolcedorme, 2267 m a.s.l.), and Calabrian Apennines, mainly siliceous, with Coastal Chain (M.Cocuzzo,1541 m), Sila Massif (Botte Donato,1929 m), Serre Calabre (M. Pecoraro, 1423 m) and Aspromonte Massif (Montalto, 1956 m). Plains are few, linked to the presence of rivers.

The climate is of Mediterranean type, with maximum precipitation during the winter and minimum in the summer and vice versa for temperature, but strong meso-climatic variations occur depending on altitude, topographic features and location respect to the sea. As consequence, the typical Mediterranean bioclimate is restricted to a belt mainly close to the coast, flowing to the European one going up to the top of mountains. Vegetation varies with bioclimate: xerophile oaks (*Quercus virgiliana, Q. suber, Q. ilex*), Mediterranean maquis (*Pistacia lentiscus, Rhamnus alaternus, Myrtus communis*, etc.) and therophytic pastures dominate the coastal thermo-Mediterranean belt; mesophile oaks and mixed woods (*Quercus cerris, Q. pubescens *s.l., *Castanea sativa, Acer *sp. pl., *Ostrya carpinifolia*, etc.) in the meso-Mediterranean hilly belt; beech woods (but also *Pinus laricio *and *P. leucodermis *woods), brooms and mountain pastures in the mountain European belt.

From the ethno-botanical point of view, Calabria is one of the most interesting region, for the dominant cultures of several people in the past (Greeks, Romans, Byzantines, Arabs, Normans etc.). Until some decades ago the road network was poorly developed and villages were isolated, so that the culture of the "subsistence" and some archaic customs were kept. Today agricultural (cereals, vegetables, grapes, olives and citrus fruits), pastoral and tourist activities characterize above all the way of life of people.

In order to make a first sampling of data in Calabria region, a preliminary ethnobotanical research was carried out both in some mountain areas and in coastal places.

In the mountain belt, data are presented for Castrovillari (foothill of Pollino Massif) and for Acri (in the upland plain of the Sila), in Cosenza district, in the northern part of the region. Other information was collected in the southern Calabria near S.Stefano di Aspromonte, Cittanova and S. Giorgio Morgeto (Aspromonte Massif), Reggio Calabria district. The cited villages are located into or near important protected areas (Pollino National Park, Sila National Park, Aspromonte National Park, Tarsia lake natural reserve).

Castrovillari and Morano are starting points of interesting excursions in the upland of the Pollino National Park (with the rare *Pinus leucodermis*), or of itineraries in canoe along torrents. Acri, S.Stefano di Aspromonte, Cittanova, S. Giorgio Morgeto are at the centre of interesting naturalistic areas where the endemic *Pinus calabrica *but also the tropical fern *Woodwardia radicans *grow.

Other data were collected in coastal or hilly areas of Crotone district (Cirò), Reggio Calabria district (Scilla) and Catanzaro district (Montauro and S.Elia).

**Figure 1:**
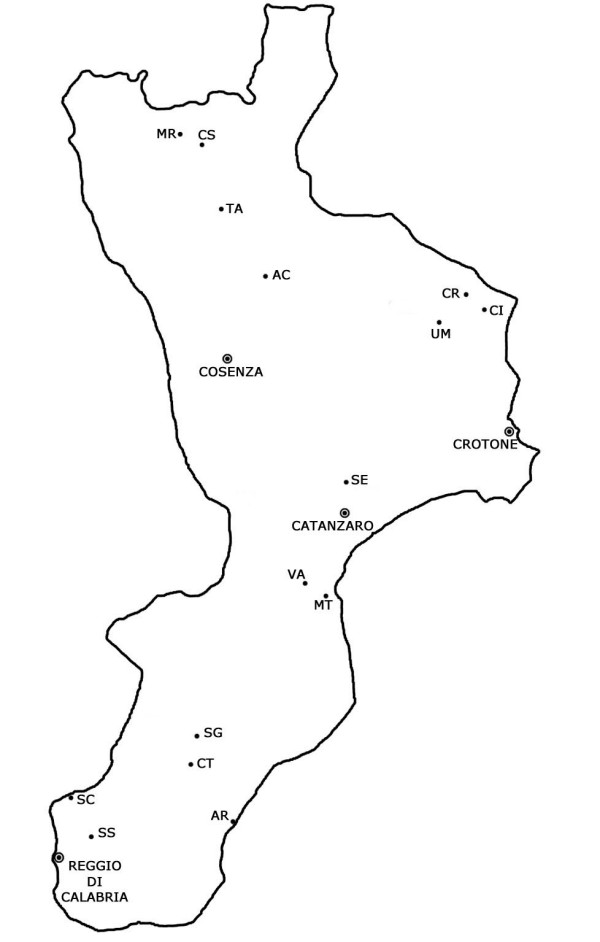
Map of the investigated areas in Calabria region. (AC Acri; AR Ardore; CI Cirò; CR Crùcoli; CS Castrovillari; CT Cittanova; MT Montauro; MR Morano; SC Scilla; SE S.Elia; SG S.Giorgio Morgeto; SS S.Stefano di Aspromonte; TA Tarsia; UM Umbriatico; VA Vallefiorita).

Brief news was also collected for Crucoli, Umbriatico (Crotone), Vallefiorita (Catanzaro); Ardore (Reggio Calabria); Morano and Tarsia (Cosenza) (Fig. 1).

The colonies of the "Magna Graecia" were above all located along the Ionian and southern coasts. The name of Scilla is associated to the sea monster that, according to the Odissea, terrorized the sailors of the Messina Strait (for the strong streams that are present in the Strait). This village is known for the fishing of the swordfish, but it lives also with agriculture and tourism. Cirò is the ancient Ypsicron (Krimisa in the Magna Graecia), now famous for the full-bodied wine.

Several internal towns date back to the presence of the Normans (e.g. Montauro) or have medioeval aspect (e.g. S.Giorgio Morgeto).

## Methods

In the "open" interviews informants (farmers, shepherds, housewives) were asked to furnish for each plant: local name, folk use (in veterinary science, as anti-parasitic agent, in the nourishment, in domestic and ritual fields), formulation and used parts, possible recipes, possible association with other plants. More than 100 informants were interviewed, mostly over 50 years old (near Cirò 5 informants were between 90 and 96 years old, others between 80 and 86 years old). Plants were identified by local informants through gathering in the area or through examination of the fresh plants which were showed them by the researchers. Cited voucher herbarium specimens are kept in the herbarium of the Università della Calabria (acronym CLU) and in the Museo Nazionale Arti e Tradizioni Popolari (Rome)(acronym Mat). Taxa are reported according to Pignatti [8]. The collected data were compared with those quoted by Gastaldo [9], with the pharmacobotanical literature of southern Italy and of the near Sicily [1-7,10-28], and with other studies cited in the text, in order to highlight possible novelties or concordances of uses.

## Results and discussion

The uses of 62 plants belonging to 34 families are reported in Table 1, 2, 3: 8 taxa are employed in veterinary science and 8 as anti-parasitic agents (Tab. 1), 19 in human nourishment (Tab. 2), 5 as seasoning and 38 for other uses (cosmesis, illegal fishing, domestic or handicraft field, agriculture, rituals) (Tab. 3). Plants are listed according to the families' alphabetical order, even inside them. Some species (4) particularly toxic for the livestock (according to the effects referred by the informants) are described in Table 4. The most represented families are: Compositae (7 species), Labiatae (7 species) and Leguminosae (4 species).

**Table 1:** Ethnoveterinary and anti-parasitic uses of plants in some areas of Calabria (Southern Italy)

Family, scientific name, local name (voucher specimen)	Used Part	Use	Preparation/Administration	N *	Locality	Habitat
APOCYNACEAE						
*Nerium oleander *L. – liantru – CLU2	Br	The plant is considered enemy of the moles, well-known eaters of roots of vegetables	According to the folk opinion the branches of oleander were stuck into the ground in order to poison the moles (use still actual).	3	CI	To, he
CAPRIFOLIACEAE						
*Sambucus nigra *L. CLU5	Fle	To attract the flies that were killed	Leaves were put in small bunches in the houses	1	CS	Ru, di,to
COMPOSITAE						
*Inula viscosa *(L.) Aiton – spulitru CLU7	Ap	Elderly people used it to eliminate the parasites of the rectum	The whole plant was inserted in the anus (veterinary use for asses and mules)	5	CI	Unc, ru, caso
*Matricaria chamomilla *L. – galumedda, camomilla	Dfh	Repellent for woodworms and other insects	They were put among the linen	2	SC,CT	Cu, ru
EQUISETACEAE						
*Equisetum telmateja *Ehrh. – stocca e ammenta CLU10	Ep	To make cow-beds for bovines, horses and sheep without evaluate the toxicity of the plant	Shepherds and herdsmen use it dry	5	CI	Di, ed-wo, da
JUGLANDACEAE						
*Juglans regia *L. – noce CLU17	Le	Anti-parasitic (above all for bugs)	Decoction (it was poured in the bed)	1	AC	Wo, di
	Le	Anti-parasitic also for furnishings, garments and pieces of furniture		1	CI	
LABIATAE						
*Lavandula angustifolia *Miller – ramaietto(MR), spigaddossa (CS)	Ft	Repellent, deodorant of linen	Picked before the complete flowering, dried and put in small bugs	6	MR, CS, MT, SE, CT, SC	Cu
*Ocimum basilicum *L. – basilico CLU18	Ep	Repellent for flies and mosquitoes	The plant is put on the windows	1	AC	Cu
LAURACEAE						
*Laurus nobilis *L. CLU22	Fr	Repellent for flies (veterinary use)	Macerate in olive oil applied onto the coat of the animal	3	MT	Tewo
*Vicia faba *L. – fava	Se	Fodder for animals		5	CI	Cu
LILIACEAE						
*Ruscus aculeatus *L. – vruscia	Br	To keep mice at a distance	They are hung in the houses	1	TA	Tewo
*Urginea marittima *(L.) Baker – cipuddazzu CLU26	Ep	Repellent and anti-parasitic agent for insect and mice	The farmers put the whole plant in granaries and silos, above all in "canizze" (containers woven of reeds) that contained broad beans of various type	5	CI	Sl
MALVACEAE						
*Malva sylvestris *L. CLU 27	Le	Gastritis	Decoction (veterinary use)	2	MT, SE	Unc, ru, edro
MORACEAE						
*Ficus carica *L. – see footnote (1) CLU28	Le	To increase the output of milk	Leaves were given as fodder to cows	5	CI	Ru, wa
RANUNCULACEAE						
*Helleborus bocconei *Ten. – aricchja CLU32	Ro	For the bronchitis of bovines. The animal would be recovered in short, and it was recognizable for the hole remained on it. Then, it seems that it would have become immune from diseases, after this remedy. No possibility of recovery existed in the case in which the disease was in advanced stage; in this case it occurred atrophy of the hole containing the 3 pieces of the stalk, then expelled.	According an ancient tradition, the cowherds of Calabria region let dry the long petiole of the basal leaves, divided into 3 parts; it was inserted in a hole practised on the back of the ear of the animal (from here the vernacular name), or under the fur of the lateral part of the neck. If the animal reacted "in positive way" to this graft, a swelling of the surrounding region developed around the stem, with a necrotic area of the diameter of approximately 1 cm, provoking a small hole on the ear, or a small cavity (on the neck).	4	CI, UM	He, mo-wo (su-cl)
SCROPHULARIA-CEAE						
*Scrophularia canina *L. – erva lupara CLU34	Ap	To treat the lameness ("pedàina") of the sheep.	Veterinary use. There is not a breeder who not used this plant for whichever problem, both in human medicine and in veterinary science. The breeders whom speak about this plant are many.	5	CI	Sa, gr, st
*Verbascum thapsus *L. – lingua e voiju CLU35	Le	Against the lameness ("pedàina") of cows	Not communicated	5	CI	Drme
SOLANACEAE						
*Cestrum parqui *L'Hér – erva fetusa CLU36	Ep	Repellent for animals	Cowherds planted it at the edges of the bushes in order to discourage the entrance of other animals, because it gives off a bad smell (poisonous plant).	3	CI	To, he, be
THYMELAEACEAE						
*Daphne gnidium *L. – junastrum, paparina ("ppè ntassari")	Ep	Used against the invasions of water snakes.	Put in lakes where domestic animals drink after the transhumance	5	CI	To, ro, cla, sa
CLU38	Ep	Some fishermen use it to capture ells of streams.	Thrown in the water	5	CI	
	Ba	To treat "papillomas" (veterinary-magical use)	A plaiting with the bark was made and then it was knot around the papilloma; the animal would be recovered in a short time.	5	CI	
URTICACEAE						
*Urtica dioica *L. – ortica CLU40	Ap	Once a mash with bread was made	By boiling (it was the only fodder for chicks of Turkey)	5	CI	Ru

* = citations

**Localities**: AC Acri; CI Cirò; CR Crùcoli; CS Castrovillari; CT Cittanova; MT Montauro; MR Morano; SC Scilla; SE S.Elia; TA Tarsia; UM Umbriatico

**Plant parts used**: Ap aerial part; Ba bark; Br branches; Dfh dry flower heads; Ep entire plant; Fle fresh leaves; Fr fruit; Ft flowery tops; Le leaves; Ro root; Se seeds

**Habitat**: Be beachs; Caso calcareous soils near to the water; Cla clayey grounds; Cu cultivated species or cultivations; Da damp areas; Di ditches; Drme dry meadows; Edro edges of roads; Edwo edges of woods; Gr gravels; He hedges; Mowo mountain woods; Ro rocks; Ru ruins; Sa sandy places (grounds); Sl slopes with rocks and silt, areas with sand and silt facing West; St stony grounds; Su sunny grounds; Sucl sunny clearings; Tewo termophile woods; To along torrents; Unc uncultivated areas; Wa walls; Wo woods.

(1) In Cirò the first ripe figs are named 'botta', those ripening in summer 'fichi'.

**Table 2:** Food uses of plants in some areas of Calabria (Southern Italy)

Family, scientific name, local name (voucher specimen)	Used Part	Use	Preparation/Administration	N *	Locality	Habitat
AMARANTHACEAE						
*Amaranthus retroflexus *L.	Ys	Food use	Picked before the flowering	1	CR	Cu, ru
BORAGINACEAE						
*Borago officinalis *L. – borragina CLU3	Le	Food use	In salad (finely minced leaves)	1	AC	Unc, pig,
	Fl	Dye for aromatic vinegars		1	AC	co
CAPRIFOLIACEAE						
*Sambucus nigra *L. CLU 5	Fl	Used in cookery	Flowers in oil to make the classic fritters ("pitte ccu majiu")	5	CI	Ru, di,to
COMPOSITAE						
*Centaurea napifolia *L. – zimurro Mat1, *C. sphaerocephala *L. – zimurro Mat2	Ap	Food use	Plants, peeled from thorns, were eaten cooked	1	AR	Be
*Silybum marianum *(L.) Gaertner – cardu marianu	Yle	Food use	In salad	2	MT, SE	Fi, unc,ru
	Ro, fh	Food use	Boiled with other vegetables	2	MT, SE	
*Taraxacum officinale *Weber – ricottedda, cicorione	Le	Food use	In mixed salad or as boiled vegetable	3	CS, MT,SE	Unc, ru, me
ERICACEAE						
*Arbutus unedo *L.	Fr	Food use	Astringent jam	2	CT, SS	Tema
FAGACEAE						
*Castanea sativa *Miller – castagno CLU12	Fr	Fruits were the main food in periods of famine for people that carried out heavy jobs	Cooked fruits	5	CI	Oawo*
JUGLANDACEAE						
*Juglans regia *L. – noce CLU17	Hu	To make a liqueur ("nocino")	Macerate in alcohol	5	CI	Wo, di
LABIATAE						
*Mentha spicata *L. – amenta Mat4	Le	Seasoning	To season dishes	1	VA	Dame
*Origanum heracleoticum *L. – rigunu CLU19	Le	To season the salad	It was collected in summer	5	CI	Drme
*Rosmarinus officinalis *L. – rosimarinu CLU20	Le	Seasoning	On meats	5	CI	Sa, tema
*Salvia officinalis *L. – sarbia CLU21	Le	Flavouring in cookery		5	CI	Drme, ru
LAURACEAE						
*Laurus nobilis *L. CLU22	Le	To obtain the "panicottu", bread seasoned with oil	As basis of broth for the "panicottu" (given to children)	5	CI	
	Le	As spice in cookery	On meats, among dried figs etc.	5	CI	
LEGUMINOSAE						
*Phaseolus vulgaris *L. – fagiuoli	Se	Substitute of the coffee	Roasted seeds	1	AC	Cu
*Spartium junceum *L. CLU23	Fbu	Food use	Eaten after preserving in vinegar (above all in the past)	2	SS, CT	
MORACEAE						
*Ficus carica *L. – see footnote (1) CLU28	Fr	Food use ("fichi 'mbottiti")	Dried figs are cut and filled with almonds, walnuts, chocolate, spices; then they are browned in oven	1	CS	Ru,wa
MYRTACEAE						
*Myrtus communis *L. – Murzìla (CS), murtidda (MR) CLU29	Br	To obtain the "tortaniddi"	White dried figs are run through partly pruned branches	2	CS, MR	Tema
ONAGRACEAE						
*Epilobium angustifolium *L. – garofanino	Ys, st	Food use	In salad	1	MT	Dame, cle (be-wo)
PLANTAGINACEAE						
*Plantago major *L. – simula	Le	Food use	Tender leaves are good ingredient for soups	1	AC	Ro, ru, da
PORTULACACEAE						
*Portulaca oleracea *L. – purchiacchia – CLU31	Yle	Food use	In salad or boiled	2	MT, SE	Cu, ru
RANUNCULA-CEAE						
*Clematis vitalba *L. – grambuntine	Ys	Food use	Boiled in soups or in omelette	1	MR	He, wo
*Ranunculus ficaria *L – favucello	Yle	Food use	In salad	2	MT, SE	Dame
	Ap	Food use	As a vegetable (soups, other dishes)	2	CT, SS	
URTICACEAE						
*Urtica dioica *L. – ardicela CLU40	Ap	Food use for children in growth (tonic treatment)	By boiling and seasoning with oil	5	CI	Ru

* = citations

**Localities**: AC Acri; AR Ardore; CI Cirò; CR Crùcoli; CS Castrovillari; CT Cittanova; MR Morano; MT Montauro; SE S.Elia ; SS S.Stefano di Aspromonte; VA Vallefiorita

**Plant parts used**: Ap aerial part; Br branches; Fl flowers; Fr fruit; Hu husk; Le leaves; Ro root; Se seeds; St stem; Yle young leaves; Ys young sprouts.

**Habitat**: Be beachs; Bewo beech woods; Cle clearings; Co cowsheds; Cu cultivated species or cultivations; Da damp areas; Dame = damp meadows; Di ditches; Drme dry meadows; Fi fields; He hedges; Me meadows; Oawo* oak woods included *Q. cerris *woods; Pig around the pigsties; Ro rocks; Roa roads; Ru ruins; Sa sandy places (grounds); Tema termophile maquises; To along torrents; Unc uncultivated areas; Wa walls; Wo woods.

(1) The small unripe fruit is named 'cuzzummeru', and when in May-June it become ripe, it is named 'botta'. In August other 'cuzzummeri' (the true figs) mature (CS).

**Table 3:** Domestic, handicraft and miscellaneous uses of plants in some areas of Calabria (Southern Italy)

Family, scientific name, local name (voucher specimen)	Used Part	Use	Preparation/Administration	N *	Locality	Habitat
ACERACEAE						
*Acer *sp. – occhjiajnu	W	To make spoons		1	CI	Ma, Mewo
ANACARDIACEAE						
*Pistacia lentiscus *L. – scinu CLU1	Fr,Ap	Ointment	Oil for lamps	5	CI	
	Ap	It was used to make brooms and during the funerals in the past	For the use in funerals the leafy branches were put between the coffin and the dead men in order to allow that the corpse could be preserved for a long time	5	CI	Tema
APOCYNACEAE						
*Nerium oleander *L.	Br	To make the sling (better with olive-tree)		1	AR	To, he
ARALIACEAE						
*Hedera helix *L. – L'edira (CI)	Le	To wash hairs (they become shining)	Infusion	1	CT	Wo, wa
CARIOPHYLLA-CEAE						
*Saponaria officinalis *L. – saponaria	Le, ro	Detergent	Plant parts were used by the farmers instead of the soap	2	MT, SE	Da, ru
CHENOPODIACEAE						
*Spinacia oleracea *L. – spinaci	Ap	To make the garments of black wool shining and bright	To rinse the garments with cooking water	1	AC	
COMPOSITAE						
*Cynara cardunculus *L. subsp. *scolymus *(L.) Hayek	Le	To make shining dark clothes	Decoction	1	AC	Cu
*Inula viscosa *(L.) Aiton – spulitru CLU7	Ap	To make brooms	It was collected by elderly people	5	CI	Unc, ru caso
*Matricaria chamomilla *L. – galumedda, camomilla	Fh	To wash blond hairs	Decoction	1	CS	Cu, ru
CORNACEAE						
*Cornus sanguinea *L. – russula, sanguinella	W	To make tools for kitchen (spoons, goblets etc.) and collars for goats		1	CI	Wo, edwo
EUPHORBIACEAE						
*Euphorbia amygdaloides *L. – tutumagghu – CLU11	La	Child practice	Children used to spread with latex wounds or mucous membranes, only to widen that part and as test of endurance of the pain. The latex provokes swelling of the sex male organ, with persistent pain	5	CI	Oawo*
FAGACEAE						
*Castanea sativa *Miller – castagno CLU12	Frb	To wash the hairs	Decoction	2	MT, SE	Oawo*
GRAMINEAE						
*Arundo donax *L. – canna CLU14	St	Stake in kitchen gardens/vineyards		5	CI	Di, edwa
	St	To make baskets (see *Olea europaea *subsp. *oleaster*)		1	CI	
HYPOLEPIDACEAE						
*Pteridium aquilinum *(L.) Kuhn – filici CLU16	Ap	It was used for its aroma by the herdsmen to wrap dairy products		5	CI	Cle,da, edro
JUGLANDACEAE						
*Juglans regia *L. – noce CLU17	Hu	To dye hairs	Infusion or decoction	5	CI	Wo, di
LABIATAE						
*Salvia officinalis *L. CLU21	Le	It was used to obtain white teeth	Leaves were rubbed on the teeth	5	CI	Drme, ru
LEGUMINOSAE						
*Lupinus albus *L. – lupino	Ep	Fertilizer	It's buried underground in the vineyards because it "would strengthen" the grapevines	1	AC	Cu, ru
*Phaseolus vulgaris *L. – fagiuoli	Se	To wash woollen and cotton coloured clothes	Decoction with pods and shelled beans to brighten up and to fix the colours	1	AC	
*Spartium junceum *L. CLU23	Br	Domestic and agricultural use	To make brooms and laces for vines	5	CI	
	Br	Domestic use	To make brooms, hides and shelters for cattle	5	SC, CT, SS, SG	
	Br	Textile use	In the first post-war period, not having available enough clothes, the branches of the broom were weaved. The plant, after gathering, was kept in the running water of torrents ("fiumare"); then it was beaten on the stones of these streams, and was dried in the sun. Successively it was combed to extract an excellent fibre, the one that the elder women wove for their family. Also bags and carpets were made with it.	5	CI	
MORACEAE						
*Ficus carica *L. CLU28	Fr ('pas-si-luni'),	Magical use: for being sure not come in contact with snakes for an entire year. If in the case, snakes would not bitten	Fruits gathered on the ground or dried to the sun were kept by grandmothers and given to eat in May 1° rigorously (this practice assured what exposed in 'use')	5	CI	Ru, wa
OLEACEAE						
*Olea europaea *L.	Br	To make the sling and spoons		1	AR	Cu
*Olea europaea *L. subsp. *oleaster *(Hoffmans & Link) Negodi – u ghjastru	Br	Baskets for bread, desserts, clothes for washing; "sporte", containers for vintage; "panàri", baskets to gather fruit, once indispensable trousseau of brides	Baskets are made together with *Arundo donax *slices and *Clematis vitalba *stems. Reeds are gathered in January and cut to strips in August. In this month *C. vitalba *and wild olive-tree young branches ("vrinchi") are collected and put in water for 2 days. The higher edges of the baskets are made with the branches of wild olive-tree.	1	CI	Ma
*Phillyrea latifolia *L.	W	To make collars for animals; good fuel	See proverbs in the text	5	CI	Ma
RANUNCULACEAE						
*Clematis vitalba *L. – viteriva	St	To make baskets (see *Olea europaea *subsp. *oleaster*)		1	CI	Wo, ma
ROSACEAE						
*Pyrus communis *L. – pero; pirajnu (the wild pear tree)	W	To make the dish ("coppa") of the poor men. Also the wood of wild pear tree was used.	A big trunk was chosen, it was divided in half along its axis and then it was carved with some tools ("gajru" and "martelletta"). This wood was very hard.	1	CI	Cu
SALICACEAE						
*Populus *sp.	W	To make collars for cows		1	CI	Edwa
*Salix *sp.	W	To make collars for cows (if poplar not was found)		1	CI	Edwa
SCROPHULARIA-CEAE						
*Verbascum thapsus *L. *V. phlomoides *L. – tassu	Le	To make wicks for oil lamps		2	MT, SE	Drme
	St	They were used to light the fire in old ovens for the bread		2	MT,SE	
UMBELLIFERAE						
*Ferula communis *L. – feddurazzu CLU39	St	To make bungs for barrels, flasks and sculptures; once it was also used by the artisans to make chairs and baskets	The dry stem is cut by the farmers. It is employed still today from the elderly in the local handicraft	2	CI	Ru, ro, unc
*Pimpinella anisum *L. – anice	Fr	They can be used as bait	Food for fishes	1	AC	Cu
URTICACEAE						
*Parietaria officinalis *L., *P. diffusa *Mert. et Koch. – erba vetriola	Ap	To clean glasses, bottles and demijohns put in pulping	To rub the aerial part with water	4	MT, SE CT, SS	
*Urtica dioica *L. – ardicela CLU40	Ap	To wash clothes and wools	Decoction	2	MT,SE	
VERBENACEAE						
*Vitex agnus castus *L. – vrigna marina CLU41	Br	Farmers utilized them to make peculiar baskets ("sporteddi")	Dry branches	5	CI	Da, sa, to
VITACEAE						
*Vitis vinifera *L. – vite	Br	Branches ("sarmienti") to soothe the pain (magical ritual)	An odd number of trimmed shoots (or their decoction) to put on the stomach of the patient	1	AC	Cu

* = citations

**Localities**: AC Acri; AR Ardore; CI Cirò; CS Castrovillari; CT Cittanova; MT Montauro; SC Scilla; SE S.Elia; SG S.Giorgio Morgeto; SS S.Stefano di Aspromonte

**Plant parts used**: Ap aerial part; Ep entire plant; Fh flower heads; Fr fruit; Frb fruit bark; Hu husk; La latex; Le leaves; Ro root; Se seeds; St stem; W wood.

**Habitat**: Caso calcareous soils near to the water; Cle clearings; Cu cultivated species or cultivations; Da damp areas; Di ditches; Drme dry meadows; Edro edges of roads; Edwa edges of water-courses; Edwo edges of woods; He hedges; Ma maquises; Mewo mesophile woods; Oawo* oak woods included *Q. cerris *woods; Ro rocks; Ru ruins; Sa sandy places (grounds); Tema termophile maquises; To along torrents; Unc uncultivated areas; Wa walls; Wo woods.

**Table 4:** Toxic plants for animals in the folk knowledges of Cirò, Calabria (Southern Italy)

Family, scientific name, local name (voucher specimen)	Toxicity	N *	Locality	Habitat
EQUISETACEAE				
*Equisetum telmateja *Ehrh – stocca e ammenta	Plant with high toxicity for animals, above all for bovines and sheep (these animals usually refuse this plant)	5	CI	To, da, di,edwo
OXALIDACEAE				
*Oxalis pes-caprae *L. – campanedda; campanelle, trifoglio delle tortore° ; erba viscida, visciola °° – C45	People referred cases of sheep that, after eating a great amount of this plant (in fields infested by the plant in flower in a percentage of 80%), died or aborted. The sick animals showed: colic, tympanitis, paralysis for the limbs, coma. The herb is harmful above all for sheep (sometimes for goats), but innocuous for bovines and horses	5	CI	Ru, cu (cla,si,su in hill)
SOLANACEAE				
Cestrum parqui L'Hér – erva fetusa C46	Cases of mortality of bovines due to the ingestion of the plant have been referred	3	CI	To, he (in low hill), be
UMBELLIFERAE				
*Ferula communis *L. – feddurazzu C47	Plant toxic for grazing animals. The stem, if dried, loses its toxicity	2	CI	Ru, to, roa, unc

* = citations ° modern name °° old name

**Localities**: CI Cirò

**Habitat**: Be beachs; Cla clayey grounds; Cu cultivated species or cultivations; Da damp areas; Di ditches; Edwo edges of woods; He hedges; Roa roads; Ru ruins; Si silt grounds; Su sunny grounds; To along torrents; Unc uncultivated areas.

### Veterinary medicine

In Calabria the breeding of animals is a very important activity and many dishes are realized e.g. with pork meat (a primary resource), or with products derived from goats, sheep and cows (pecorino, ricotta, mozzarella etc.) mainly in the hilly and mountain areas.

A particularly in-depth research was carried out near Cirò (Crotone).

The plants described in this section are mainly of clearings of oak woods, chestnut and mixed woods (*Helleborus bocconei*), garrigues and maquises (*Daphne gnidium*), meadows (*Inula viscosa, Malva sylvestris*), gravels, sandy and stony grounds (*Scrophularia canina*).

#### *Helleborus bocconei*

In the past, since the first years of XX century, it was the only remedy known by the cowherds in case of bronchitis of bovines. This species, toxic as fresh plant due to poisonous substances (glycosides elleborin and elleborein, and some alkaloids), loses its toxicity after drying [29]. *H. bocconei *is named "aricchja" in Cirò, "radicchia" in other localities of Calabria [4], "radicchia" or "raricchia" in Sicily, where the subsp. *siculus *is used to diagnose and cure the pneumonia of cattle [22,26]. The gathering occurred on Friday and only in those places that because of their geographical position faced either at the sea and at the mountain. People thought that this procedure exalted the curative properties of the plant. We don't know if this is true, but it is a sure thing that this species is an excellent remedy, so that it still survives in the most internal rural areas of Calabria. Its "secret" still hands down from father to son. An analogous use is documented for other areas [30].

#### *Scrophularia canina*

The use as an antiseptic, anti-inflammatory and cicatrising in veterinary science of *S. canina *(a medicinal plant for excellence in the Crotone district) is reported for some regions of central Italy [30-33]. In Calabria this practice is still now alive.

#### *Cestrum parqui*

This subspontaneous ornamental species is rather unusually used as arepellent for animals; its unpleasant smell probably represents probably a guard signal against more serious effects (being a toxic plant for cattle).

#### *Inula viscosa*

It was used to eliminate the parasites of the rectum in asses and mules. Bernardo [3] reports the cicatrising use of this herb.

#### *Daphne gnidium*

The use for papillomas seems to be more a magic remedy than a medical one.

### Anti-parasitic uses

The reported plants are cultivated (*Juglans regia*), or species growing in the Mediterranean maquis (*Laurus nobilis, Nerium oleander*) and garrigues (*Daphne gnidium, Urginea maritima*). Some species are still now employed.

*Nerium oleander *is a plant dear to the farmers and very requested, since considered enemy of the moles, known eaters of roots and vegetables. In Cirò, planting branches of this plant is still now a means to kept out holes. This fact could perhaps be explained through the branches eated by these animals, which after die because of the poison. The use is not cited for other regions, but in Sicily the flowers are spread on the ground of areas infested with cockroaches [22].

#### *Lavandula angustifolia*

The presence of this plant in the Pollino Mountain and other areas of the region can account for its common use as repellent agent.

Among other repellent agents: *Juglans regia *leaves for bugs, *Ocimum basilicum *for mosquitos and *Laurus nobilis *fruits (macerate in olive oil) for flies, put on the coat of cattle [34]. *Sambucus nigra *branches were hung in rooms to attract flies, then captured.

A particular still practised use is that of *Urginea maritima *bulb, as a repellent for insects and mice in granaries, silos and containers of broad beans [13]. In Sicily this bulb is analogously used as a repellent for mice [21] or as rat poison [22].

The memory of the anti-parasitic use of *Delphinium consolida *is kept in the vernacular name of Cirò: "erba ppè pidocchi" (herb for louses).

### Human nourishment

The plants reported in this section grow above all in meadows (*Borago officinalis, Origanum heracleoticum, Plantago major*), ruderal areas (*Amaranthus retroflexus*), edges of roads (*Silybum marianum*), woods (*Castanea sativa*), clearings of wood (*Epilobium angustifolium*), open environments (*Spartium junceum*) and in the Mediterranean maquis (*Myrtus communis*). Even some species are gathered on beachs (*Centaurea napifolia, C. sphaerocephala*). Some cultivated species were used for peculiar purposes (e.g. roasted seeds of *Phaseolus vulgaris *as a substitute of the coffee in Acri, upland of Sila). Almost all the described species are still employed nowadays in Calabria, except for the more thorny species (*Centaurea *sp. pl.).

Calabrian people resorts in the nourishment to a lot of vegetables like the aubergine, with properties useful to reduce the amount of cholesterol in the blood [35]. Like in other regions, several species are eaten, e.g, *Borago officinalis, Taraxacum officinale, Urtica dioica*. Flowers of *Sambucus nigra *("maju") are fried to make classic fritters ("pitte ccu majiu"). The young leaves of *Ranunculus ficaria*, the only edible plant of the Ranunculaceae family (apart from *Clematis vitalba *cooked buds), are eaten in salad, but also in soups. Among the less common food uses we cite that of *Amaranthus retroflexus *young buds [15,26], a ruderal species gathered in Cirò. This use is cited for Calabria region also by Picchi and Pieroni [6],that reported another species of *Amaranthus, A. lividus*, as food plant. Uncommon is also the food use of *Plantago major *(tender leaves in soups), described in the upland of Sila near Acri.

Some thorny plants (Carduae, e.g. *Silybum marianum*) are eaten also in the near Basilicata region [15,26], while the food use of *Centaurea napifolia, C. sphaerocephala *and *Spartium junceum *pickled buds, not cited elsewhere, is probably linked with the extreme poverty of past periods. *S. junceum *should have some toxicity for the presence of cardioactive principles. The use of young buds and pith of *Epilobium angustifolium *in salad is also new.

Contributions on wild food plants of Calabria region were made by Bernardo [3], by Picchi and Pieroni [6] and by Nebel et al. [7]. Bernardo [3] reports the use of *Asphodelus fistulosus *roots, *Leopoldia comosa *bulbs, "qepez", and *Tordylium apulum *leaves, in addition to *Asparagus acutifolius, Chenopodium bonus-henricus, Cichorium intybus, Clematis vitalba*. Picchi and Pieroni [6] highlight particularly the food use of *Allium triquetrum *(kept in olive oil after boiling in water and vinegar), of *Lythrum salicaria *(young buds in salad, stem without cortex boiled in vinegar or in olive oil), of *Hypochoeris glabra, Lotus edulis *(leaves and fruits), *Chrysanthemum segetum *(the more gathered species in Aspromonte), *Reseda alba *and other wild herbs.

Among the seasoning herbs, we cite *Myrtus communis *whose branches are utilized to make small spits to which figs are skewered for a winter eating (see also [3]). An analogous unpublished use was described in the Tyrrhenian area of Basilicata [36]. Also *Mentha spicata, Laurus nobilis *and *Origanum heracleoticum *are used as seasoning, together with *Salvia officinalis *(this last species in Calabria is subspontaneous in dry meadows and ruderal areas). The dye properties of *Borago officinalis *flowers are exploited for aromatic vinegars. Some practices cited in other Calabrian papers (e.g. that one of *Ficus carica *cinder in order to preserve seasoned salami)[5] have not been found by us.

### Domestic and handicraft uses

In Cirò the use of *Spartium junceum *to make clothes, sacks and carpets dates back to the first decade of XIX century, as testified by some elderly men born in the first years of the XX century, whose parents were devoted to this work. In the Graecanic area the textile use of this broom was particularly practised in Aspromonte, e.g. in Bova [37] and near some Albanian minorities. At the present, it survives in few Calabrian countries (e.g. Serrastretta) [38] and Cerzeto [39] and in some folkloristic events [40]. It is also well documented in the Museum of. S.Paolo Albanese (Basilicata region) [3]. Several fabrics (knapsacks, blankets, towels, napkins etc.) from Calabria made with *Spartium junceum *are kept in the Museum of Arts and Folks Traditions (Rome); they were collected at the beginning of the XX century.

In Calabria the basketry is now particularly practised in San Giorgio Morgeto, Delianuova, San Roberto and Crucoli, but above all in Soriano Calabro [41]. From the inventories of the Museum of Arts and Folks Traditions (Rome) result that some Calabrian baskets were made with *Fagus sylvatica, Ulmus minor *and *Abies alba, Arundo *sp.pl., *Salix *sp., straw, but also small containers ("fiscelle") were made with brooms and other baskets ("nasse") with reeds and *Juncus *sp. The use of *Salix caprea *branches is documented for the Mt. Pollino area [3].

Moreover, in this paper we report the use of the branches of *Vitex agnus castus *– typical plant of riverbeds and edges of torrents ("fiumàre") – to make baskets ("sporteddi") near Cirò. This use is known since ancient times: in fact, the first term of the scientific binomial *Vitex agnus castus *(*Vitex*) means flexible shoot to bend, from the Latin "vieo" [42] (= to bend, to interwoven). This last word corresponds to the Greek "lìgos" (used also by Omero = Italian agnocasto) [8], with its verb "ligòo"(= to bend, to interwoven) [43] because – according to Dioscoride – the branches of this plant are long and pliable [44]. Rocci [43] writes that *Vitex agnus castus *(also called αγνος) is named in Italian "agnocasto" but also "vetrice". This term "vetrice" is attributed to *Vitex agnus castus *by Palazzi [45] too, while Zingarelli [46] calls it "a willow for baskets". In the dialects of central-southern Italy the word "vetrice" corresponds to some species of *Salix *[15,31,32]. Therefore *Vitex agnus castus *and *Salix *sp. pl. are called with the same term since their branches are used to bend.

The use for baskets of *Vitex agnus castus *is undescribed in the current Italian ethnobotanical papers, included the enormous work of Atzei [47]. Lieutaghi [48] writes that the Latins interwove its branches as those of a willow and that the plant is used in southern areas (of the France, where the willows are rare) to make baskets. Pirone [49] reports that the priestesses of Cerere slept on pallets interwoven with its branches.

Also *Ferula communis *is still used for this purpose and to make rustic chairs, as it happen in Sicily [23,24]. Other species are employed for brooms (*Pistacia lentiscus, Inula viscosa*,etc.), while there are some memories of wicks for oil lamps made with *Verbascum phlomoides *and *V. Thapsus*. An oil for lamps was obtained from *P. lentiscus *[5].

Two trees furnished the matter of various Calabrian artefacts (from the inventories of the Museum of Arts and Folks Traditions, Rome): *Fagus sylvatica *(chests for storaging bread, baskets, cradles) and *Citrus bergamia *(snuff-boxes)[50]. The very original Calabrian craft is art of the shepherds" (that engrave the wood) of the Serre and Sila Greca should be described from an ethnobotanical point of view. Hand looms with *Fagus sylvatica *wood are still made in Cariati (Cosenza) and Castelsilano (Crotone) [51]. Some plants are used in Cirò to make collars for animals (*Phyllirea latifolia, Populus *sp., *Salix *sp., *Cornus sanguinea*) and tools for kitchen (*Cornus sanguinea*). Elderly people says in Cirò with regard to *Phyllirea latifolia *wood: "Liternu lignu eternu", that is "*P. latifolia *wood is eternal due to its hardness when it is dried". Another proverb says: "Liternu ppe focu e ppe mprnu", that is "*P. latifolia *wood to make a fire and for the hell", since it burns much.

In the Calabrian economy of subsistence, a discreet number of plants were used in decoction to brighten up the colour of clothes: *Spinacia oleracea, Cynara scolymus *and *Urtica dioica *(leaves), in addition to *Phaseolus vulgaris*. Hanging the thorny *Ruscus aculeatus *in the houses, in order to keep the rats away, is perhaps magical; or it could be a residual of the use to wrap cheeses or ropes (these last ones employed to hang cheeses to the ceiling).

### Other uses

A few plants are used in the cosmetic field: e.g. *Hedera helix *and *Matricaria chamomilla *in order to hair dye, and *Abies alba*, whose twigs are used in decoction to prepare deodorant footbaths. A limited number of plants is described also in agriculture: *Arundo donax *as a 'stake', *Lupinus albus *as a fertilizer for ground, *Spartium junceum *for laces in vineyards and vegetable gardens.

### Toxic plants

Among these we can cite *Daphne gnidium*, called "junastrum" (ginestraccio, that is bad broom) but also "paparina ppè ntassari" (that is a plant that sleeps in order to poison) – 10 fruits can kill a men [52]. The fishes caught with this system (illegal fishing) are always more or less toxic for men [53]. Toxic plants for cattle are cited in Table 4 (the information was collected near Cirò). Among these, *Oxalis pes-caprae *introduced from South-Africa. The excessive consumption of this plant provokes intestinal inflammations, blood in the urines and often death by collapse in ovines, bovines and horses [54], due to the high amount of calcium oxalate. Milk cows were infected in 1818 by the "Morbo Ignoto" (unknown disease), also named "Pinzanese". It was treated with vinegar, salt and rubbing human dung [55].

Other toxic plants for animals are *Ferula communis *(oleoresins, resins)[56], *Cestrum parqui *(parquine, solasonine), *Equisetum telmateja *(silica and thiaminase). The folk name of this last plant is "stocca e ammenta" (that is you divide and unite) because the different parts of the plant can be detached and again inserted on the stem.

### Plants and vernacular names

Some vernacular names derive from the culture of the "Magna Graecia" or from the following Bizantine rule. *Pistacia lentiscus *is named "scinu", "scine", which comes from the Greek "schinus". This last word comes from "to cut through, to carve", because the bark of the mastic tree is cut throughout to bleed the mastex [44]. *Plantago major *is called "peltinervia". This vernacular name results from "pentinervia" (from the Greek "pentà" = 5), whereas in several regions of Italy it is called "cinquenervia". Regarding the shape of the leaves, *Verbascum thapsus *is called "lingua 'e voiju" (that is tongue of ox), while *Adonis annua *"eriva bedda" (erba bella, that is beautiful herb) for the peculiar red colour of the corollas. *Cestrum parqui *is named "erva fetusa" (that is stinking herb) for its unpleasant smell. The sap of the vine is poetically called "pianto della vite" (the tears of the vine).

## Conclusion

The preliminary reported data – in comparison with those from other Calabrian ethno-botanical papers – show that a big work is still to carry out in this region, where each village is "an island" for the past geographical difficulties of communication and for the above described culture of "subsistence". These data can appear fragmentary, because found out in the course of many years – in various stages – but they contribute to rebuild some plugs of a very rich patrimony in the past.

From the research it emerges that the practice or the memory of the veterinary, food, anti-parasitic, cosmetic, agricultural and domestic uses are still alive near the inhabitants of the investigated areas of Calabria region, particularly for the food uses. Among these practices, some are curious but consolidated, e.g. those for veterinary and anti-parasitic purposes, and worthy of further scientific investigation.

This recovery can have relapses in the ethno-pharmacological field, but also in the handicraft, economic and tourist sector. The preservation of traditional knowledge e.g. in the food or artisan fields may be source of some income in local enterprises.

This research is also offered as a contribute to the knowledge of the ethno-biological roots of the investigated region.

## Competing interests

The author(s) declare that they have no competing interests.

## Authors' contributions

The field work for data collection was carried out above all by GDF and in a limited way by the other authors. Data analysis and manuscript preparation were conducted by all authors, but above all by PMG. Scientific coordination was carried out by NGP.
